# Lead acid battery recycling for the twenty-first century

**DOI:** 10.1098/rsos.171368

**Published:** 2018-05-16

**Authors:** Andrew D. Ballantyne, Jason P. Hallett, D. Jason Riley, Nilay Shah, David J. Payne

**Affiliations:** 1 Department of Materials, Imperial College London, Exhibition Road, London SW7 2AZ, UK; 2 Department of Chemical Engineering, Imperial College London, Exhibition Road, London SW7 2AZ, UK

**Keywords:** electrochemistry, lead-acid batteries, recycling

## Abstract

There is a growing need to develop novel processes to recover lead from end-of-life lead-acid batteries, due to increasing energy costs of pyrometallurgical lead recovery, the resulting CO_2_ emissions and the catastrophic health implications of lead exposure from lead-to-air emissions. To address these issues, we are developing an iono-metallurgical process, aiming to displace the pyrometallurgical process that has dominated lead production for millennia. The proposed process involves the dissolution of Pb salts into the deep eutectic solvent (DES) Ethaline 200, a liquid formed when a 1 : 2 molar ratio of choline chloride and ethylene glycol are mixed together. Once dissolved, the Pb can be recovered through electrodeposition and the liquid can then be recycled for further Pb recycling. Firstly, DESs are being used to dissolve the lead compounds (PbCO_3_, PbO, PbO_2_ and PbSO_4_) involved and their solubilities measured by inductively coupled plasma optical emission spectrometry (ICP-OES). The resulting Pb^2+^ species are then reduced and electrodeposited as elemental lead at the cathode of an electrochemical cell; cyclic voltammetry and chronoamperometry are being used to determine the electrodeposition behaviour and mechanism. The electrodeposited films were characterized by scanning electron microscopy (SEM) and X-ray photoelectron spectroscopy (XPS). We discuss the implications and opportunities of such processes.

## 1. Introduction

**Figure 1.**
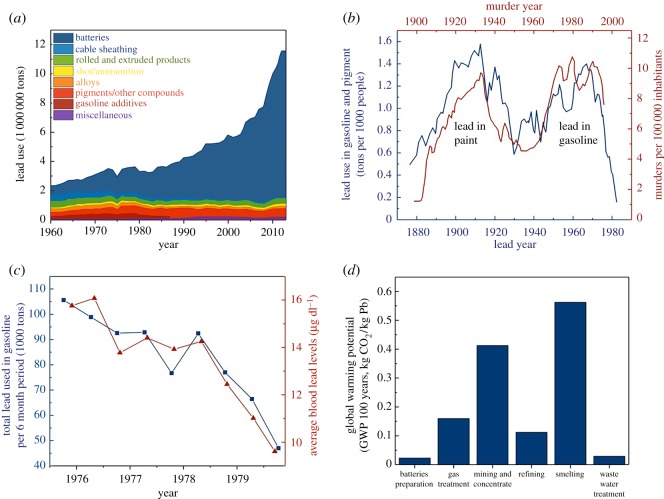
(*a*) Global applications of lead from 1960 to 2014 (reproduced from Davidson *et al.* [2]), (*b*) historical lead use in pigment and gasoline contrasted with annual murder rate in the USA (reproduced from Nevin [3]), (*c*) parallel decreases in blood lead values and the amounts of lead consumed in gasoline between 1976 and 1980, in the USA (USEPA/Environmental Criteria and Assessment Office 1986; [4]) and (*d*) cradle-to-gate results for lead production life cycle inventory in terms of global warming potential (reproduced from Davidson *et al.* [2]).

Lead and lead-containing compounds have been used for millennia, initially for plumbing and cookware [1], but now find application across a wide range of industries and technologies [2]. Figure 1*a* shows the global quantities of lead used across a number of applications including lead-acid batteries (LABs), cable sheathing, rolled and extruded products, ammunition, alloys, pigments and gasoline additives during the latter part of the twentieth and beginning of the twenty-first centuries. A general trend of decreasing lead use occurred for most applications since the 1980s with the exception of LABs. The consumption of lead through the production of LABs increased from 0.6 Mt of lead in 1960 to 10 Mt in 2012, when it accounted for greater than 85% of lead used [2]. This increase was due to two factors, the increased number of automotive vehicles and so-called ‘deep cycle’ LABs which are popular for standby and emergency power supply, with automotive LABs accounting for 75% and deep cycle LABs 25% of the sector. LABs are popular, particularly in the automotive sector, because the chemistry is mature, robust and well understood and they can deliver the high, initial burst of power necessary for the starter ignition of internal combustion engines [2]. It is also worth noting that LABs are still present in state-of-the-art hybrid and fully electric vehicles due to their position as ‘the*’* energy storage device for the 12 V internal electronics [5].

Despite the widespread application, the use of lead is not without its problems and limitations. Lead is a highly poisonous material affecting almost every organ in the body, with the nervous system the most seriously affected by the toxicity of lead, in both children and adults [6]. Long-term exposure can result in decreased cognitive performance in tests that measure functions of the nervous system. This can lead to behavioural problems, a learning deficit and lower IQ. Infants and children are particularly susceptible as they have a disproportionate exposure to toxins, possess immature metabolic pathways, will progress through sensitive developmental growth periods and have a greater period of disease manifestation [7]. Nevin [3] detailed how societal lead exposure has significant correlation with teen pregnancy, assault, rape, robbery and murder rates in society. The use of lead in paint pigments and gasoline in particular was shown to have a strong societal influence. Figure 1*b* shows the tons of lead used in the US per 1000 inhabitants (blue) and the annual murder rate (red) with the *x*-axis offset by 20 years between datasets. Two peaks in the lead use plot can be observed that correspond to the use of lead pigments in paint (1890–1930) and the use of gasoline in lead (1950–1980). There are two corresponding peaks in the annual number of homicides per 100 000 people (murder rate) that occur 20 years later that have a two- and fivefold increase in the murder rate. While a number of factors are likely to influence the murder rate, the correlation between the two is striking and the influence of lead as a neurotoxin is well understood. From a more positive perspective, the USEPA/Environmental Criteria and Protection Office documented (figure 1*c*) the decrease in lead used in gasoline on blood lead levels from 1976 to 1980, during which time the lead used in gasoline decreased by 57% with a corresponding 40% reduction in average lead blood levels [4].

**Figure 2.**
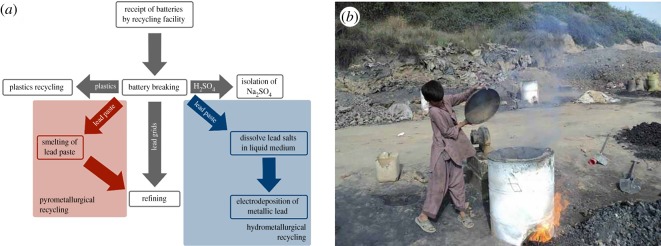
(*a*) Flow diagram showing pyrometallurgical and hydrometallurgical lead recycling process and (*b*) child in India smelting lead in an informal recycling operation. Reproduced with permission from pureearth.org [8] (Copyright 2016 Pure Earth).

With the increasing production of LABs it is of vital importance that contamination of the environment is minimized at their end of life by an effective recycling system. Recycling of LABs is one of the great success stories for the recycling industry with up to 98% of the lead-acid battery able to be recycled. Pyrometallurgical processing dominates industrial lead recycling; a typical process flow diagram is shown in figure 2. Initially, the spent LABs undergo battery breaking, in which batteries are shredded so that their constituent parts can be separated: lead paste, lead grids, dilute sulfuric acid and polypropylene or polyethylene plastic casings. The shredded plastic and sulfuric acid are easily separated from the lead-containing products which are insoluble and have a much larger density. Once washed, the plastic is fed into conventional plastics recycling and neutralized sulfuric acid isolated as CaSO_4_ or Na_2_SO_4_. The lead grids and paste can be separated from each other through sieves with the metallic lead grids passed directly into a refining operation to remove alloy impurities. The lead pastes consist of a mixture of PbSO_4_, PbO, PbO_2_, Pb_2_O_3_ and metallic Pb. PbSO_4_ is initially converted to PbCO_3_, followed by smelting of the entire mixture to reduce all of the Pb-containing compounds to metallic Pb. Smelting is a high-temperature operation, typically operating at 1100–1300°C, where metallic lead can be recovered via reduction with carbon powder. Once the lead paste has been reduced to metallic Pb it can then be refined with the metallic lead grids [9].

Despite its success, there are still a number of drawbacks of the pyrometallurgical Pb recycling process, primarily related to operational and environmental concerns [10]. Smelting has a high energy demand due to the high operating temperatures, while the use of carbon as a fuel leads to the generation of CO_2_. The high energy demand in conjunction with the production of CO_2_ means that lead smelting has a comparatively high global warming potential (GWP). Figure 1*d* shows a breakdown of the GWP of primary lead manufacture. While these values will be different than those for recycled (secondary) lead, two processes are present in each manufacturing source: smelting and refining. Smelting has a GWP of approximately 0.55 kg CO_2_ (kg Pb)^−1^ compared to refining which has a GWP of approximately 0.12 kg CO_2_ (kg Pb)^−1^, highlighting the impact of lead smelting [2]. In addition to the release of CO_2_ from oxidation of coke, there are other highly toxic emissions from the smelting process termed metal hazardous air pollutants (HAPs). Metal HAPs are predominantly composed of compounds of lead, antimony, arsenic and a small amount of other metal compounds. Lead compounds constitute approximately 70% by weight of HAP emissions [11], which, when considering the health implications discussed earlier, is a significant potential source of harm to the environment, particularly for those living and working in the facility and the immediate vicinity. Methods do exist to prevent particulates and hazardous gases from release into the environment including bag houses, wet scrubbers and hoods [11], although these need to be well maintained to ensure effective performance. However, eliminating smelting from lead recycling entirely could enable a process where the emission of particulates was greatly reduced, and follows the modern principles of pollution prevention which prioritize avoidance of end-of-pipe treatment. In addition, the use of measures to minimize environmental release does not reduce the risks to those that work in the facility.

The risks associated with LAB recycling are accentuated for those working in low to middle income countries (LMICs). Table 1 shows [8] the most polluting industrial process worldwide when ranked by disability adjusted years of life lost (DALY) [8]. One DALY can be thought of as 1 year of ‘healthy’ life lost, or alternatively a measure of the difference between health in the current situation and one where the population was able to age free of disease and disability [12]. By this measure LAB recycling is the most polluting industrial process, resulting in between 2 000 000 and 4 800 000 DALYs. While rates of lead exposure and release are carefully controlled in developed countries, in LMICs they can be considerably higher. For example, Moturi and co-workers [13] assessed the surface concentration and blood lead level of workers at a lead smelter in Kenya. All measurements of contamination were above levels permitted by the U.S. Occupational Health and Safety Administration, even those taken from office areas. Blood lead levels from the Kenyan lead smelter were between 43.4 and 62.2 µg dl^−1^, values over three times higher than those in figure 1*c*, where gasoline additives were used in petrol. The comparatively low blood lead levels in figure 1*c* were linked to significant increases in teen pregnancy, assault, rape, robbery and murder rates and thus the implications of higher blood lead levels are clear. The elevated levels in Kenya were attributed to inadequacies in engineering controls, work practices, respirator use and personal hygiene. In addition, exposure risks at more informal facilities are even higher. Figure 2*b* shows an informal smelting operation in India where the smelting operation is carried out in the open air with no personal protective measures while coloured emissions can be seen, suggesting the release of Pb HAPs. One study by Haefliger *et al.* investigated a community in Dakar, Senegal where informal LAB recycling was taking place [14]. Eighteen children died in five months from a rapidly progressive central nervous system disease, and in follow-up studies of adults and children living in the same area a range of blood levels from 39.8 to 613.9 µg l^−1^ with a mean of 129.5 µg l^−1^ were measured. Surrounding areas were also heavily contaminated [15]. Ericson *et al.* studied informal lead recycling across 90 LMICs estimating that there were up to 29 241 sites across these countries, where up to 16.8 million people were exposed to elevated lead levels [16].

**Table 1.** Most polluting industrial processes worldwide, arranged by disability adjusted years of life lost (DALYs). Reproduced with permission from pureearth.org [8] (Copyright 2016 Pure Earth).

rank	industry	DALY
1	used lead-acid battery recycling	2 000 000–4 800 000
2	mining and ore processing	450 000–2 600 000
3	lead smelting	1 000 000–2 500 000
4	tanneries	1 200 000–2 000 000
5	artisanal small-scale gold mining	600 000–1 600 000
6	industrial dumpsites	370 000–1 200 000
7	industrial estates	370 000–1 200 000
8	chemical manufacturing	300 000–750 000
9	product manufacturing	400 000–700 000
10	dye industry	220 000–430 000

While the success of LAB recycling is encouraging, it is clear that significant improvements can be made in both energy consumption and exposure minimization. To this end, there have been significant efforts to replace the smelting operation with either a so-called hydrometallurgical electrowinning one [9] or leaching followed by low-temperature calcination. In leaching followed by low-temperature calcination the lead is dissolved into solution then recovered selectively through precipitation. The recovered compound(s) can then be calcined to produce a high surface-area mixture of Pb compounds that can be used directly as the ‘leady oxide’ material LAB manufacture [9]. In hydrometallurgical lead recycling, the reduction of the salts in lead paste occurs though a solution-based methodology. An example of a hydrometallurgical recycling process is shown in figure 2, where the recovery of the plastic, lead grids and sulfate occurs through conventional means and the lead paste undergoes wet processing where it is dissolved in a solvent followed by electroreduction, also known as electrowinning, through the application of a cathodic potential to produce metallic Pb. These deposits can be high value as they are obtained in high purity at temperatures close to room temperature. An electrochemical process such as this has many potential benefits in that the technique is performed at relatively low temperature, eliminates the mechanism by which lead particulates are produced during smelting and the process can be stopped very quickly, significantly reducing operational safety concerns [10]. Furthermore, decarbonization of the electrical power input can lead to a very low overall emissions process. However, lead salts, particularly oxides, have poor solubility across a variety of different solvents with PbO, PbO_2_ and PbSO_4_ being insoluble in water. To overcome the poor solubility, initial efforts focused on the use of strong acids such as HBF_4_ and HSiF_6_; however, these processes were limited by significant safety concerns and high cost [17].

Recently, there have been studies of two more benign solvents. Acidic aqueous brines were demonstrated to have moderate solubility of lead salts with the process capable of dissolving up to 25 g l^−1^ lead. These brines were found to improve solubility by complexation and reaction with the acid and chloride present, as demonstrated in equations (1.1)–(1.3). Once the Pb salts were dissolved in solution, metal salt impurities were removed by galvanic exchange with metallic Pb. Pb was then subsequently recovered through the electrolytic reduction with the half-cell equations for the cathodic and oxidation processes detailed in equations (1.4) and (1.5). This was called the PLACID process for which development was led by Téchnicas Reunidas, Spain. Its promise led to the award of an EU FP6 grant for its commercialization with the development of a pilot plant capable of producing 400 kg of pure lead per day [17,18]

1.1 $$\begin{array}{c} \text{PbO}+\text{2HCl}+\text{2NaCl}\to \text{N}{\text{a}}_{2}\text{PbC}{\text{l}}_{4}+{\text{H}}_{2}\text{O}, \end{array}$$

1.2 $$\begin{array}{c} \text{Pb}+\text{Pb}{\text{O}}_{2}+\text{4HCl}+\text{4NaCl}\to \text{2N}{\text{a}}_{2}\text{PbC}{\text{l}}_{4}+2{\text{H}}_{2}\text{O}, \end{array}$$

1.3 $$\begin{array}{c} \text{PbS}{\text{O}}_{4}+\text{4ChCl}\to \text{N}{\text{a}}_{2}\text{PbC}{\text{l}}_{4}+\text{N}{\text{a}}_{2}\text{S}{\text{O}}_{4}, \end{array}$$

1.4 $$\begin{array}{c} \text{cathode:}\;\text{ PbC}{\text{l}}_{2}+2{\text{e}}^{-}\to \text{Pb}+2\text{C}{\text{l}}^{-} \end{array}$$

1.5 $$\begin{array}{c} \;\text{and}\;\text{anode:}\;{\text{H}}_{2}\text{O}\to 2{\text{H}}^{+}+\;1\;/\;2\;{\text{O}}_{2}+2{\text{e}}^{-}. \end{array}$$

The most significant hydrometallurgical process to date is that by Aquametals, who developed a proprietary process using an aqueous solution of methylsulfonic acid and EDTA. The inclusion of EDTA was demonstrated to enable a solubility of lead paste of 75 g l^−1^, so increasing production capacity per unit volume of solvent. Aquametals is now a successful growing company with a market capitalization of $247M as of July 2017. Their success demonstrates the commercial practicality of hydrometallurgical processes in the recycling of LABs [19].

**Figure 3.**
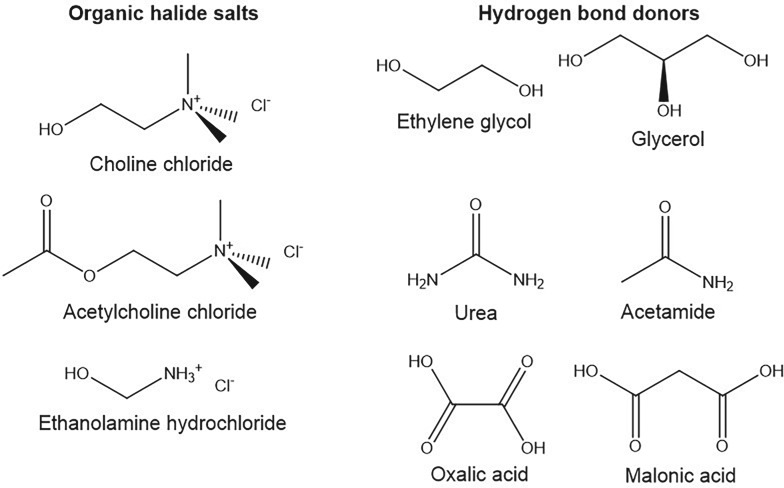
Example structures of deep eutectic solvents.

All hydrometallurgical processes developed to date have relied on the use of strong acids to improve the solubility of the respective lead salts. However, a novel class of solvents have emerged recently called deep eutectic solvents (DESs) that demonstrate high solubility of metal salts despite having little associated hazards [20]. DESs are liquids formed from a eutectic mixture of Lewis/Brønsted acids and bases which may contain a variety of cationic, anionic or neutral species. A number of different types of DES exist that are classified based on their composition and the nature of the interaction between the components. Type III DESs, in particular, have received considerable attention. These are liquids formed from a combination of a tetra-alkylammonium halide salt and small polar organic molecule, commonly referred to as hydrogen bond donors (HBDs). Some example components are shown in figure 3, where common salts include choline chloride (ChCl), acetylcholine chloride and ethanolammonium chloride, while common HBDs include amide, polyol and acetate organic groups. An example DES is the liquid formed between one molar equivalent of ChCl and two molar equivalents of urea. ChCl melts above its decomposition point of 302°C and urea melts at 133°C, yet when mixed together the two form a liquid with a melting point of 12°C. It was previously thought that the suppression of melting point was primarily due to complexation of the halide anion by the HBD; however, recent density functional theory calculations suggest that competitive cation/anion HBD interactions govern the eutectic formation [21].

Because of their composition (approx. 4.6 M halide content and high concentration of HBD) DESs are excellent for solvating metal salts. This, combined with their electrochemical and thermal stability, makes them ideal candidates for many metal processing applications including electroplating [22], galvanic coating [23], electropolishing [24] and metal extraction [25]. The high solubility and electrochemical processability extends to lead-containing salts. In the Payne group, we have previously investigated the recycling of lead based hybrid organic inorganic perovskites (HOIPs) using the DES Ethaline 200, a 1 : 2 molar mixture of ChCl and ethylene glycol (EG) [15]. It was demonstrated that after dissolution, 99.8% of the lead could be electrochemically removed from the solvent demonstrating this was a viable recycling route for HOIPs [15]. Wang *et al.* studied the reduction of a solid pellet of PbO using direct electrochemical deoxidation in the same liquid, observing that it was possible to obtain close to a 100% reduction to metallic Pb [26]. Additionally, Sun *et al.* studied the electrochemical properties of PbSO_4_, PbO and PbO_2_, the components of lead paste, in the DES Reline 200, a 1 : 2 molar mixture of ChCl and urea, finding that electrochemical behaviour remained fairly consistent between the respective Pb salts [27].

Presently, we are undertaking a study of lead recycling that builds on our and others' research in the development of a new recycling process for LABs using the unique properties of DESs. Funded by the UK Engineering and Physical Sciences Research Council (EPSRC) and working in conjunction with Enviowales, a commercial lead recycler in the UK, the project is called ‘Recycling Lead-Acid Batteries' or RELAB. We seek to take a holistic and multidisciplinary approach to the development of the new process, considering novel scale-up strategies, cell design and comparative energy balances of new and existing processes to develop cutting edge fundamental research with direct implications for the commercial sector. Here we detail some preliminary results regarding chemistry choice and development, focusing on the use of the DES Ethaline 200.

## 2. Material and methods

### 2.1. Deep eutectic solvent and lead solution preparation

Choline chloride (Aldrich, 99%) and EG (Aldrich, +99%) were mixed together by stirring and heating at 60°C until a homogeneous, colourless liquid had formed, known industrially as Ethaline 200. To this was added PbCl_2_ (Aldrich, greater than or equal to 98%). The concentration of all solutions were 5 mM unless otherwise stated.

### 2.2. Electrochemical measurements and bulk deposition

Cyclic voltammetric (CV) and chronoamperometric (CA) measurements were performed on an Ivium compactstat controlled by Iviumsoft software v. 2.121. A three-electrode set-up was used comprising a 3 mm diameter. Pt disc working electrode (W.E.) (BASi) polished with 50 nm alumina (Buehler), Pt gauze counter electrode (C.E.) and Ag/Ag^+^ pseudo-reference electrode (R.E.) because of the high Cl^−^ concentration.

For electrodeposition experiments, an identical three-electrode set-up was used as for the CV and CA measurements described above. Pt foil was polished with 4 and 1 µm diamond paste followed by 50 nm alumina. The polished foil was cut into 10 × 5 mm squares and affixed to a 0.25 mm diameter. Pt wire with a small section of conducting carbon tape. The back side of the electrode, including the carbon tape, was protected with kapton tape such that during the experiment the only electrically conducting contact with the electrodeposition solution was that of the Pt foil. Deposition was performed for 600 s at a potential of −0.42 V versus Ag wire pseudo-reference electrode.

### 2.3. Solubility measurements

To 5 ml of Ethaline 200 was added 0.2 g of PbSO_4_ (Aldrich, 98%), PbCO_3_ (Aldrich, 99.99%), PbO (Aldrich, 99.999%) or PbO_2_ (Aldrich, 99.998%) followed by heating to 80°C with magnetic stirring for 24 h. The liquid was allowed to cool for 24 h; 1.5 ml of the Pb-containing solutions with suspended Pb salts were transferred to Eppendorf tubes and placed in a VWR MiniStar silverline microcentrifuge in which they were rotated at 6000 r.p.m. for 20 min. The liquor was decanted and its mass accurately measured. This was converted into a volume from the literature density values (1.12 g cm^−3^) [20]. The mass was typically 1.1 g. The Pb-containing Ethaline 200 solutions were then diluted to 10 ml with aqueous 1 M HNO_3_. A series of dilutions to 100 ml and 1000 ml were also prepared. Pb reference samples of 0, 1, 5, 10 and 20 mM were prepared through dilution of a 1000 ppm Pb in 1 M HNO_3_ reference (Aldrich) which were used to standardize measurements. All diluted samples were measured and solubility taken from the sample with a concentration between 1 and 20 ppm Pb. Quoted solubility values are given as the concentration measured multiplied by the dilution factor for that sample. ICP-OES was measured on a Thermo-scientific iCAP 6000 using the supplied its iTEVA software.

### 2.4. Characterization of bulk deposits

The Pb deposits on Pt foil were characterized with SEM and XPS. SEM images were recorded with a Zeiss Sigma 300 FE-SEM using the secondary electron detector operating at an accelerating voltage of 5 kV. Images were recorded using the secondary electron detector. XPS was recorded on a Thermo Fisher K-alpha operating at 2 × 10^−9^ mBar base pressure. The system was equipped with a monochromated Al K*α* X-ray source microfocused to a spot size of 400 µm. The detector was a 180° double focusing hemispherical analyser with a two-dimensional detector. Samples were mounted on conducting carbon tape. The data were manipulated using the inbuilt Avantage software.

## 3. Results

### 3.1. Solubility of lead salts in the deep eutectic solvent Ethaline 200

Of critical importance to any hydrometallurgical recycling process is that a sufficient level of the metal salts can be dissolved into the processing medium. Depending on process step, Pb recovered from LABs may contain PbSO_4_, PbCO_3_, PbO and PbO_2_. Here we have used ICP-OES to measure the concentration at saturation of the components of spent LAB paste in the DES Ethaline 200, a combination of 1 : 2 molar equivalents ChCl and EG, at room temperature with the results shown in table 2. ICP-OES is a popular method for measurement of metal concentrations due to its ability to measure a wide range of concentrations, a number of metals simultaneously and, as the liquid medium is converted into a plasma, measurements are dependent solely on the metal ion concentration and do not vary based on speciation [28]. There are significant variations in the solubility of each of these compounds with PbSO_4_ exhibiting the lowest at 1786 ppm. This is threefold lower than PbCO_3_. Al Nashef *et al.* reported that Na_2_CO_3_ had appreciably higher solubility than the corresponding chloride and bromide salts in Ethaline 200 [29], which could explain the high solubility of carbonate. No comparable sulfate solubility data could be found to offer direct comparison. Both PbO and PbO_2_ have solubilities close to or greater than that of PbCO_3_. These values are much larger than that of the oxides of the first row transition metals where solubilities ranged between 1 and 470 ppm depending on the metal and oxidation state of study [28].

**Table 2.** Solubility at room temperature of lead salts in the DES Ethaline 200 as measured by ICP-OES.

lead salt	concentration (ppm)
PbSO_4_	1786 ± 66
PbCO_3_	5872 ± 519
PbO	4453 ± 10
PbO_2_	12 186 ± 1042

The overall aim of the RELAB project is to develop a chemistry and methodology for the recovery of lead salts from spent lead waste using the unique properties of DESs. EXAFS studies of dissolved Pb salts show that they predominantly form low coordinate, anionic, chloride species in Ethaline 200 [23,30]. Indeed, previous work in the group used EXAFS to demonstrate that a variety of hybrid inorganic–organic lead halide perovskites all form four coordinate [PbCl_4_]^2−^ species [15]. Given the variety of Pb compounds in spent LABs and the dominance of chloro species of dissolved Pb salts, early investigations of Pb electrorecovery have been limited to that of PbCl_2_.

### 3.2. Cyclic voltammetry of Pb ions in the deep eutectic solvent Ethaline 200

**Figure 4.**
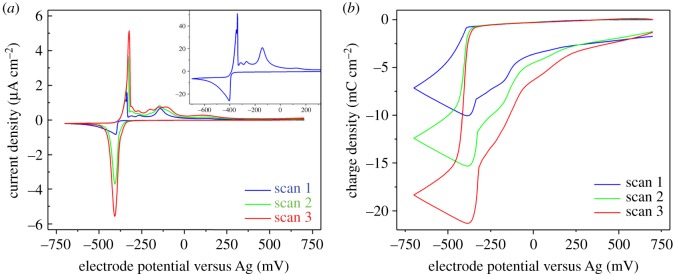
(*a*) Current and (*b*) charge versus potential plots of cyclic voltammograms of 5 mM PbCl_2_ in Ethaline 200 on a 3 mm Pt disc W.E., *ν* = 20 mV s^−1^, Pt gauze C.E. and Ag wire pseudo-R.E. at 800°C.

The CV behaviour of PbCl_2_ in the DES Ethaline 200 was studied on a Pt disc working electrode at 80°C with voltammograms for three successive scans shown in figure 4*a*. The first scan (inset) showed a distinctive deposition peak at approximately −0.4 V (versus Ag) on the cathodic scan with an onset of −0.39 V and a typical diffusion limited response. The subsequent anodic scan exhibited an initial oxidation peak with current onset of −0.37 V followed by a sharp decrease in the current. This is consistent with a system in which the rate of Pb oxidation is faster than the rate at which the Pb^2+^ ions can be complexed and dissolve/diffuse causing a perturbation of the composition at the electrode surface, blocking additional oxidation from taking place until there is a relaxation of the oxidized material. After this a second peak occurred at approximately −0.14 V that may be due to continued oxidation of metallic Pb at the electrode once the film had relaxed sufficiently. This is consistent with the behaviour observed by Abbott *et al*. [31]. On subsequent scans, a complex response was observed. The peak reduction current in scans two and three were much larger than that during the first scan, the onset potential was shifted positive by 0.1 V and the deposition current decreased to values lower than that limited by diffusion in the first scan. One scan takes 150 s and it is likely that this is insufficient for all of the oxidized Pb to dissolve from the electrode surface, resulting in an increased density of Pb ions at the electrode surface during the cathodic sweeps in the second and third scans.

For clarity, the total charge consumed is plotted w.r.t. potential in figure 4*b*, where the cathodic deposition results in an increasingly negative value for the charge density. Winand classified Pb as belonging to ‘normal’ metals from the perspective of electrodeposition, possessing very high exchange current densities associated with fast, reversible electrode kinetics [32]. This would result in Faradaic behaviour with matching cathodic and anodic charge. This is not the case in figure 4*b*, with the anodic charge having a value 80% of the cathodic charge on the first scan. In addition, an increase in the cathodic charge is observed with each successive scan. These complementary pieces of evidence support a model in which oxidized Pb^2+^ ions have limited dissolution rates into the bulk electrolyte, so this forms a resistive film on the surface that remains during the anodic sweep until a reducing potential is met. When the resistive film forms, this prevents some of the deposited Pb from being oxidized because of the inability of coordinating ligands to reach the metallic lead.

The voltammetric behaviour of PbCl_2_ in Ethaline 200 is complex. However, from the perspective of an electroreduction process, it is an ideal candidate due to the rapid nucleation and deposition exhibited on the first scan.

### 3.3. Nucleation mechanism of Pb electrodeposition in the DES ethaline 200

Nucleation and growth of metallic nuclei during the early stages of electrodeposition can have important ramifications on the properties of bulk electrodeposits including particle size, density and roughness [22]. CA measurements are often used to probe nucleation, where mathematical models are used to describe the time-dependent current profile, such as that described by Scharifker and Hills [33]. Their simple model described two limiting mechanisms of nucleation; instantaneous and progressive. Where instantaneous nucleation occurs in a CA experiment, nuclei form in the early stages after which the nuclei number remains fixed with sustained deposition occurring through growth of the existing nuclei. In the case of progressive nucleation, new nuclei form at all stages through the electrodeposition experiment. Each formed nucleus defines its own diffusion zone, thus a system which constantly forms new nuclei will produce a different shape in the current response when compared with a system where nuclei only form during the initial stages of experiment with the mathematical models for each response provided in equations (3.1) and (3.2), where *I* is the current at a given time *t* of a given CA measurement, *I*_m_ is the peak deposition current and *t*_m_ is the time at which the current maximum occurred.

3.1 $$\frac{I^{2}}{I_{m}^{2}}=\frac{1.9542}{t/t_{m}}{\left\{1-\text{exp}\;\left[-1.2564\left(\frac{t}{t_{m}}\right)\right]\right\}}^{2}\;\text{instantaneous nucleation}$$

and

3.2 $$\frac{I^{2}}{I_{m}^{2}}=\frac{1.2254}{t/t_{m}}{\left\{1-\text{exp}\;\left[-2.3367{\left(\frac{t}{t_{m}}\right)}^{2}\right]\right\}}^{2}\;\text{progressive nucleation}$$

**Figure 5.**
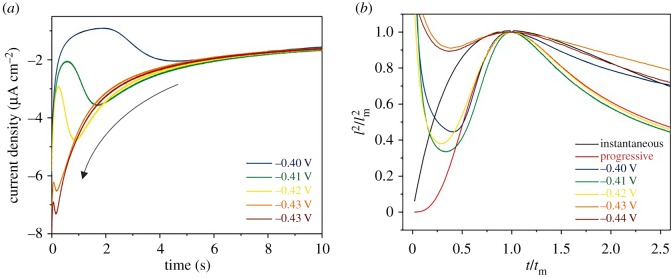
(*a*) current transients for chronoamperometry and (*b*) reduced variant plots for increasing potential of 5 mM PbCl_2_ in Ethaline 200 on a 3 mm Pt disc W.E., Pt mesh C.E., Ag wire R.E. at 80°C.

Figure 5*a* shows the CA response on a Pt surface referenced to an Ag wire pseudo-reference electrode across potentials from −0.40 to −0.44 V. In each case a peak in the current profile is seen with the position occurring at lower timescales where higher overpotentials exist, showing that nucleation and growth occur at increasingly faster timescales at more cathodic potentials.

These CA responses were then plotted against models for instantaneous and progressive nucleation, shown in figure 5*b*. The nucleation profile is often split into three stages. Stage 1 describes the initial formation and growth of nuclei on the substrate surface (*t*/*t*_m_ < 1), stage 2 describes the continued growth on nuclei and overlap of diffusion zones (*t* ≈ *t*_m_) and stage 3 the subsequent growth of the metal layer (*t* > *t*_m_). In the example shown here, two distinct responses can be observed. From −0.40 to −0.42 V progressive nucleation was observed that showed good agreement with the models across all stages. However, at more reducing potentials, where the initial nucleation occurs readily, a switch to an instantaneous response was observed. This is contrary to the behaviour of PbSO_4_ in a DES formed from ChCl and urea, as reported by Sun and co-workers [27], where instantaneous nucleation was observed across all potentials studied.

### 3.4. Properties of Pb electrodeposits

**Figure 6.**
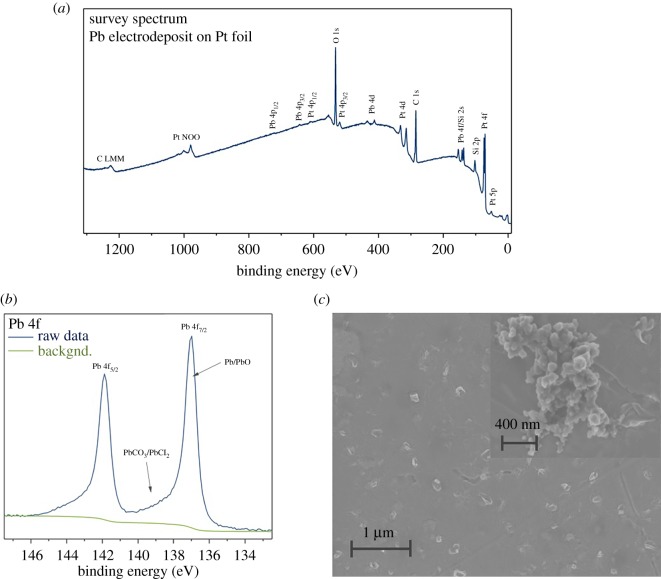
(*a*) Survey, (*b*) peak-fitted Pb 4f XPS spectra and (*c*) secondary electron SEM image of Pb electrodeposited onto a 5 × 10 mm Pt foil at −0.42 V for 600 s at 80°C, with inset an image of a Pb aggregate formed on the same sample. Referenced to an Ag wire pseudo-R.E., Pt mesh C.E.

As proof of concept of Pb electrorecovery, a potential of −0.42 V was applied to a Pt flag working electrode using a three-electrode set-up otherwise identical to that of the electrochemical studies described above. The use of a Pt flag offers consistency of substrate with those above as well as offering contrast for XPS measurements. A deposition time of 600 s was chosen so as to enable extra Pb deposition over and above that deposited during the CA experiments in §3.3. Figure 6*a* shows the XPS survey spectrum for the resulting sample. Photoelectrons from the 4f, 4d and 4p core shells of Pb indicating the presence of a Pb electrodeposit on the surface. In addition to Pb, signals are observed from the Pt substrate, carbon, oxygen and silicon. Previous studies have found that Pb produces dendritic deposits, tending to form this structure due to its fast reduction kinetics [32]. Extreme care was taken when washing electrodeposits in order to minimize potential loss of Pb; however, this also creates risk of contaminants. A weak signal relating to the loss of an electron from the 2p_3/2_ and 2p_1/2_ orbitals of chloride between 195 and 199 eV [34] was observed indicating that only small quantities of chloride remained on the sample. However, significant peaks for both carbon and oxygen were observed, suggesting the presence of some organic contaminants.

A high-resolution spectrum of the Pb 4f core level is shown in figure 6*b*, where peaks corresponding to the Pb 4f_7/2_ and Pb 4f_5/2_ core levels can be observed at 137.0 and 141.0 eV with a splitting between the peaks of 4.9 eV matching well with the literature values [35]. Pb XPS is complex as peak position is not the only variable when quantitatively fitting core level spectra, with line width, peak shape and the presence of satellites dependent on the compound of study. As such, further work is required to provide a full quantitative analysis. However, it is likely that the major contributors to the Pb 4f spectrum come from metallic Pb and PbO as labelled. Both Pb and PbO have relatively low binding energies of 136.6 for Pb and 137.5 eV for PbO [36] which match well with the highest intensity regions of the Pb *4*f_7/2_ spectrum. In addition, a weaker Pb *4*f_7/2_ signal is present at high binding energy values between 138 and 140 eV; this may be due to PbCO_3_ formation, binding energy 138.5 eV [37], through reaction of PbO with atmospheric CO_2_ and/or the presence of residual PbCl_2_ [36].

Figure 6*c* shows an SEM image of the surface of the Pb on Pt electrodeposit. Small nuclei can be observed growing from the surface which appear as small decorations between 80 and 200 nm in diameter. CA measurements recorded at −0.42 V showed deposition occurs under progressive nucleation at the early stages of nucleation. Under such a mechanism a range of nuclei sizes would be expected. In addition to regularly spaced nuclei, larger and irregular features were also observed. Figure 6*c* (inset) shows a high-resolution SEM image of one of these features. Aggregates form composed of a large number of much smaller nuclei that are likely to have formed during the electrodeposition experiment. The one shown here is much larger than the Pb nuclei with a diameter of between 600 and 1000 nm.

## 4. Conclusion

With the increasing popularity of LABs and the catastrophic health implications of lead exposure, it is increasingly important that safe and efficient recycling exists. While LAB recycling is successful, the current methods rely on the energetically expensive and potentially hazardous smelting process. To minimize safety risks and environmental contamination it is essential to include end-of-pipe treatment. In LMICs, where informal recycling operations operate and fewer environmental and safety regulations are in place, the health implications are severe, leading to LAB recycling being classified as the most polluting industrial process worldwide.

Hydrometallurgical processes, where the smelting operation is replaced with one where the Pb-containing salts are processed in solution, offer a method to minimize the release of contaminants. Typically, hydrometallurgical processes depend on the use of strong acids to solvate sufficient quantities of lead. Because of their unique properties, DESs promise comparable processing capacity of conventional processes while being composed of environmentally benign materials.

Here we have detailed early investigations of the use of the DES Ethaline 200 and its suitability as the solvent in a Pb recycling process. The proposed process involves the dissolution of Pb salts into the DES Ethaline 200. Once dissolved the Pb can be recovered through electrodeposition and the liquid can then be recycled for further Pb recycling. Pb salts show moderate solubility in Ethaline 200 ranging from 1786 ppm for PbSO_4_ to 12 186 ppm for PbO_2_. Electrochemical studies show that Pb deposits via a kinetically fast, diffusion-limited process with an onset potential of −0.39 V, though the anodic dissolution mechanism is complicated, probably related to the poor solubility of Pb salts. Chronoamperometry studies show that at close to the reduction potential Pb deposits via a progressive nucleation mechanism, whereas at more cathodic potentials a switch to an instantaneous mechanism was observed. A proof of concept deposition process was demonstrated through the deposition of Pb onto Pt foil at −0.42 V w.r.t. Ag/Ag^+^, where XPS analysis demonstrated the presence of metallic Pb on the surface with the presence of some surface PbO and PbCO_3_. From these measurements it was clearly determined that DESs are a promising solvent technology with which to recycle lead (from LABs). Future work will explore the scalability, economics and life cycle environmental impact and compare these with incumbent processes.

## Data accessibility

Data available from the Dryad Digital Repository: http://dx.doi.org/10.5061/dryad.26077 [38].
